# Epidemiology of *Acinetobacter baumannii*: analysis of hazard factors associated with positivity cases in Guizhou province, China from 2015 to 2023

**DOI:** 10.3389/fpubh.2025.1592783

**Published:** 2025-07-03

**Authors:** Zhongzhi Liang, Di Tian, Yanlan Huang, Shanjiang Dou, Anxin Yang, Zuyi Chen

**Affiliations:** ^1^Department of Laboratory Medicine, Affiliated Hospital of Zunyi Medical University, Zunyi, Guizhou, China; ^2^Department of Medical Laboratory, Qianxi People's Hospital, Qianxi, Guizhou, China

**Keywords:** *Acinetobacter baumannii*, epidemiological characteristics, Guizhou province, correlation, multi-drug resistance

## Abstract

**Introduction:**

The widely prevalent *Acinetobacter baumannii* is a gram-negative opportunistic pathogen, with the enormous potential to trigger multiple concurrent diseases. Due to its multidrug resistance, *A. baumannii* has emerged as a key monitored pathogen of WHO for surveillance in healthcare settings, particularly in intensive care units (ICUs).

**Methods:**

To identify factors associated with *A. baumannii* infection in Guizhou province, China from 2015 to 2023, a retrospective cross-sectional analysis was carried out with the data from hospital records, electronic medical databases. Fisher’s exact test (gender) and Chi-square were used for statistical tests, while *p*-values was used to define the statistically significance of variables.

**Results and discussion:**

Out of 460,620 patients; there were 6,944 positive infection events, with ICU-related infections accounting for 45.77%. Males had a significantly higher risk of *A. baumannii* infection compared to females, and host factors such as gender and increasing age were associated with greater susceptibility. In addition, infection rates were higher during warmer summer months, suggesting a possible seasonal trend in transmission. Within the population undergoing antibiotic therapy, only polymyxin B demonstrated an alarmingly high level of resistance, whereas other clinically employed antibiotics were invalid. In summary, the statistical data from these years emphasizes that gender and age are main drivers of *A. baumannii* outbreaks in Guizhou, and follow-up public health interventions need to target these factors to enhance infection control and reduce the spread. Also, the observation of high levels of resistance to multiple antibiotics is a matter of concern, as it presents significant challenges in terms of treatment.

## Introduction

*Acinetobacter baumannii* is a globally prevalent opportunistic pathogen known for causing a wide range of healthcare-associated infections (1, 2). The viability of *A. baumannii* is remarkably robust, enabling its prolonged persistence in humid environments, as well as formidable resistance to desiccation, ultraviolet radiation, and a wide range of disinfectants, like lodophor, benzalkonium chloride and chlorhexidine (3–8). *Acinetobacter baumannii* primarily contributes to nosocomial infections, particularly patients in ICUs, those with compromised immune systems diseases, individuals on long-term broad-spectrum antibiotic therapy, and patients undergoing invasive medical procedures (9–12). Simultaneously, *A. baumannii* has developed mechanisms to evade the host immune response, facilitating rapid colonization and proliferation within the host (13, 14). The global mortality rate associated with *A. baumannii* infection is alarmingly high, ranging from 30% to as high as 75%, depending on the severity of the infection and the underlying health status of the patient (15, 16). The high incidence rate and mortality caused by *A. baumannii* pose a significant public health concern, necessitating stringent control measures to curtail its transmission.

Pneumonia is the most prevalent respiratory tract infection complications caused by *A. baumannii* (17–19). Prolonged immobilization, and utilization of mechanical ventilators contaminated with *A. baumannii* is a risk factor of pneumonia for patients admitted to the ICUs, common symptoms include fever, persistent cough, and respiratory distress. Thus, it is of paramount importance to implement regular disinfection protocols for medical devices, conduct bacterial testing, employ antibacterial coatings on medical equipment, and enhance disinfection plans to effectively control respiratory diseases caused by *A. baumannii* (20, 21). *Acinetobacter baumannii* is capable of inducing urinary tract infections such as cystitis, pyelonephritis, and prostatitis. Common symptoms include increased frequency of urination, urgency, dysuria, and lower back pain in affected individuals (22, 23). The infection of the central nervous system highlights the robust immune evasion mechanism employed by *A. baumannii*, despite its lack of flagellar motility assistance (24–26). When infection occurs in the central nervous, it frequently results in the development of meningitis and brain abscess in patients, accompanied by symptoms such as headache, fever, vomiting, neck stiffness, and even disturbances in consciousness or fatality. Meanwhile, *A. baumannii* is also a significant contributor to various environmental susceptibility diseases, such as postoperative wound infections and bacteremia in healthcare settings (27, 28).

Clinical management of *A. baumannii* infections is complicated by its broad antibiotic resistance (29, 30). The formulation of specific disease prevention policies depends on the epidemiology of regional diseases, and the selection of regions is typically guided by choosing representative areas as key references. For instance, regions with inadequate medical resources or those that are excessively healthy may lack sufficient representativeness. To investigate the current status of *A. baumannii* epidemic prevention and control in China, this study selected data recorded in the Guizhou region from 2015 to 2023 for analysis. The Guizhou region, situated in a central geographic location with relatively mid-level medical care, serves as a representative sample for this research. It has been previously reported that the incidence rate of *A. baumannii* infection is higher in summer and among male patients (31). In this study, we extended the analysis beyond season and gender to include age and other factors. Additionally, we evaluated the effectiveness of antibiotics in existing hospitals, aiming to establish a more comprehensive regional trend of *A. baumannii* epidemiology.

## Materials and methods

### Data sources, inclusion criteria and case definition

The study population comprised patients hospitalized or admitted to intensive care units (ICUs) across Guizhou Province with a confirmed diagnosis of *A. baumannii infection* from January 2015 to December 2023. Surveillance data of *A. baumannii* infection in both public and private hospitals in Guizhou province obtained from the National Health and Medical Big Data Western Center of Guizhou Province were retrospectively analyzed. It is a comprehensive medical information database that stores historical hospital admission cases, diagnostic and therapeutic information, as well as special disease cases from across 12 provinces in western China (including Guizhou province). Inclusion Criteria (positive case definitions): the microbiological testing clinic provides comprehensive reports on the presence of *A. baumannii*, wherein blood and sputum are collected to detect the content and abundance by qRT-PCR. Exclusion criteria: patients with incomplete data, repeated samples, or colonization without infection excluded. Biochemical parameters: negative oxidase, positive catalase, negative indole, non-assimilation of sugars and nitrate non-reduction of submitted samples were detected by API-20NE (32). Molecular parameters: *β*-lactamase (also known as cephalosporinase) coding gene *AmpC*, is encoded in the chromosomes or plasmids of *Enterobacteriaceae* or *Pseudomonas aeruginosa*, which was also used for accurate quantitative assessment of *A. baumannii* load in clinical cases by real-timepolymerase chain reaction (Forward primer: 5′-TAAACACCACTATGTTCCG-3′/Reverse primer: 5′-ACTTACTTCAACTCGCGACG-3′. Reaction procedure: 95°C 1 min; [95°C 10 s; 60°C 25 s; 72°C 30 s] × 40 circles) (33). Post biochemical and molecular analyses, bacteria were detected after cultivation of both sputum and blood samples as a confirmed case.

### Definition of variables

Time-oriented variables, such as the year and the season (quarterly), were generated by categorizing cases according to dates of illness onset. In addition, socio-demographic factors, such as gender and age were identified. Sex was recorded as male or female, while age is divided into four groups (0–14, 15–47, 48–63, and ≥64 years). To assess the risk of infection of *A. baumannii* among patients with severe preexisting conditions or acute severe injuries, a comparative analysis of infection rates between ICU and non-ICU cases was conducted. As an independent factor, the efficacy of different antibiotic types has also been evaluated in relation to clinical treatment success and microbiological clearance capability.

### Statistical analysis

All characteristics were presented as categorical variables and summarized using frequencies or percentages. The comparison of variables between condition and outcome was conducted using Fisher’s exact test (gender). Chi-square was used for categorical variables (age and season). *p-*values less than 0.05 were considered statistically significant. Time series seasonality plots (a line graph summarizing cases by year and month throughout the study period) were drawn to show trends of cases over time. To quantify the strength of the association between the explanatory and outcome variables, we computed the odds ratios apply to binary outcomes along with its corresponding 95% confidence interval and *p*-value. Both data analysis and visualization were conducted using GraphPad Prism 8.0 software.

## Results

### Trend and seasonality of *Acinetobacter baumannii* incidence from 2015 to 2023

**Figure 1:**
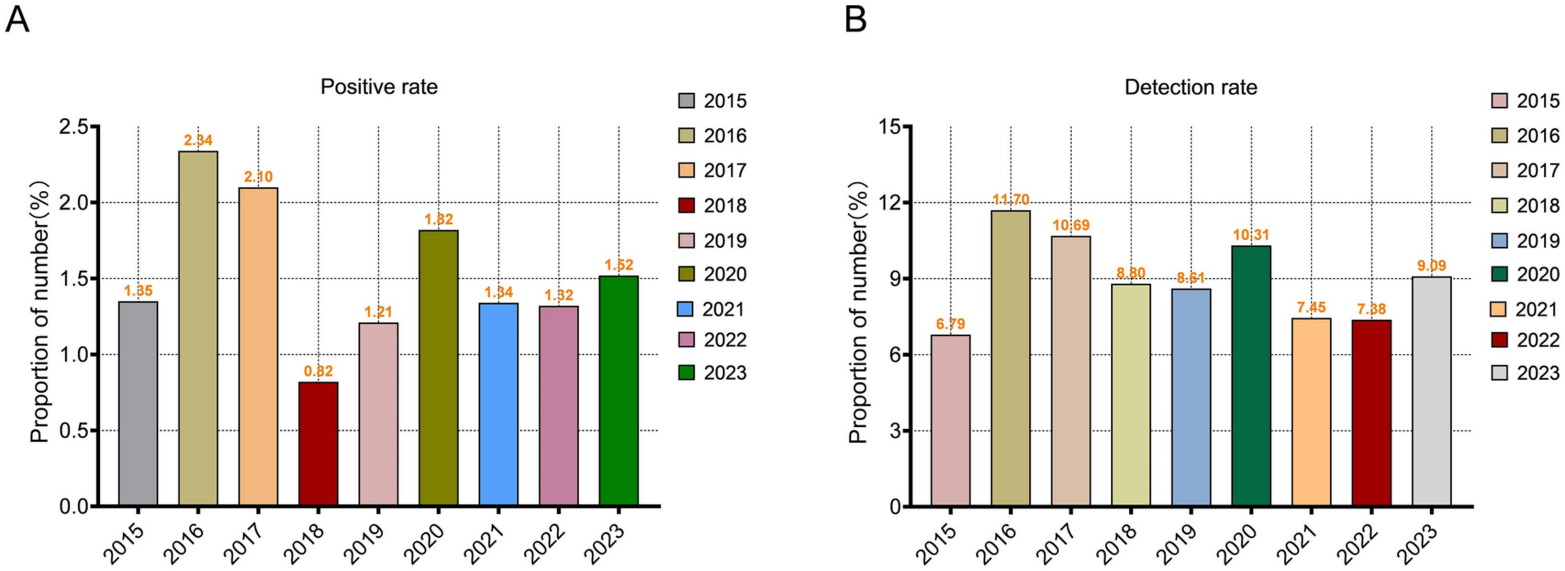
Temporal evolution of *Acinetobacter baumannii* Epidemiology in Guizhou Province from 2015 to 2023. **(A)** Changes in the positivity rate of *A. baumannii* from 2015 to 2023. **(B)** The detection rate of *A. baumannii* in hospital bacteria examination from 2015 to 2023.

A total of 6,944 positive cases of *A. baumannii* infection were identified among 460,620 tested hospitalized patients in Guizhou, resulting in an overall positivity rate of 1.51%. Case reporting was higher during the 2023 (14.12%) within 9 years, while positive rate was higher during the 2016 (2.34%). The detection rate of *A. baumannii* accounted for 8.95% of the total positive bacteria (among the pathogens identified in patients diagnosed with bacterial infections), indicating its high likelihood of being detected during infection or rapid proliferation as a relatively abundant pathogen upon detection (Figures 1A,B).

**Figure 2:**
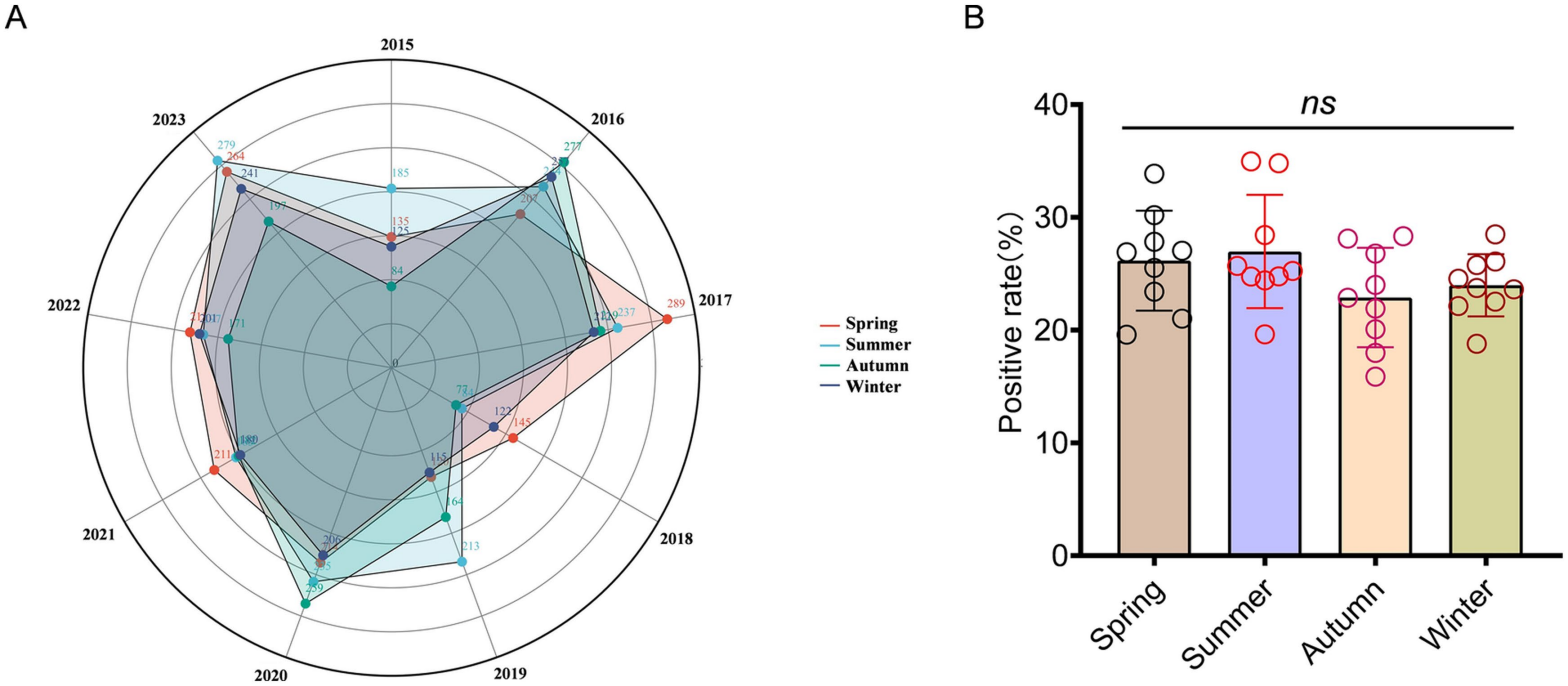
Seasonal distribution patterns of patients infected with *A. baumannii*. **(A)** The radius of a circle represents the number of positive patients, while solid circles of varying hues symbolize different seasons. **(B)** The distribution of seasonal groups among patients infected with *A. baumannii.* In **(B)**, data were shown as mean ± standard deviation. Differences were assessed using two-tailed *t*-test.

There was a predominance of high positive infection cases observed during spring and summer (autumn in 2016 and 2020), while the incidence rate remained relatively low during autumn and winter. For instance, in 2015, the proportion of infections during summer constituted 34.97% of the total infection count, whereas infections during autumn accounted for merely 15.88%. Statistical analysis of variance was conducted to examine the incidence of *A. baumannii* across different seasons. However, revolving around the variable of season, there was no statistically significant difference in the incidence rate (*p* = 0.764; Figures 2A,B). The phenomenon of inconspicuous seasonal statistical analysis results may be attributable to the limited size of the dataset or regional climatic variations. With average positive cases in spring was 200 (95% Cl: 155 to 244), summer was 207 (95% Cl: 164 to 249), autumn was 181 (95% Cl: 128 to 234), and winter was 184 (95% Cl: 144 to 225).

### Socio-demographic characteristics

Among the positive patients, there were 4,842 males and 2,102 females, resulting in a male to female ratio of 2.3:1. To examine the significant association between infection rate and gender, we performed a Fisher’s exact test. The overall results indicate a statistically significant difference in *A. baumannii* infection rates (case counts) between males and females (*p* < 0.001). Among them, the average annual number of infections in males was 538 (95% Cl: 431 to 645), in females was 234 (95% Cl: 172 to 295). The annual statistical data was presented in Table 1. This result indicated that there was a higher susceptibility among males.

**Table 1:** Trends in the male-to-female ratio of *Acinetobacter baumannii* infection in Guizhou province from 2015 to 2023.

Year	Male cases [N, %]	Female [N, %]	Total (N)	*p*-value
2015	407 (76.9%)	122 (23.1%)	529	0.000
2016	706 (71.7%)	279 (28.3%)	985	0.001
2017	687 (71.8%)	270 (28.2%)	957	0.000
2018	307 (71.7%)	121 (28.3%)	428	0.000
2019	453 (74.0%)	159 (26.0%)	612	0.001
2020	587 (64.2%)	327 (35.8%)	914	0.000
2021	533 (70.3%)	225 (29.7%)	758	0.000
2022	478 (61.3%)	302 (38.7%)	78	0.001
2023	684 (69.7%)	297 (30.3%)	981	0.001

**Figure 3:**
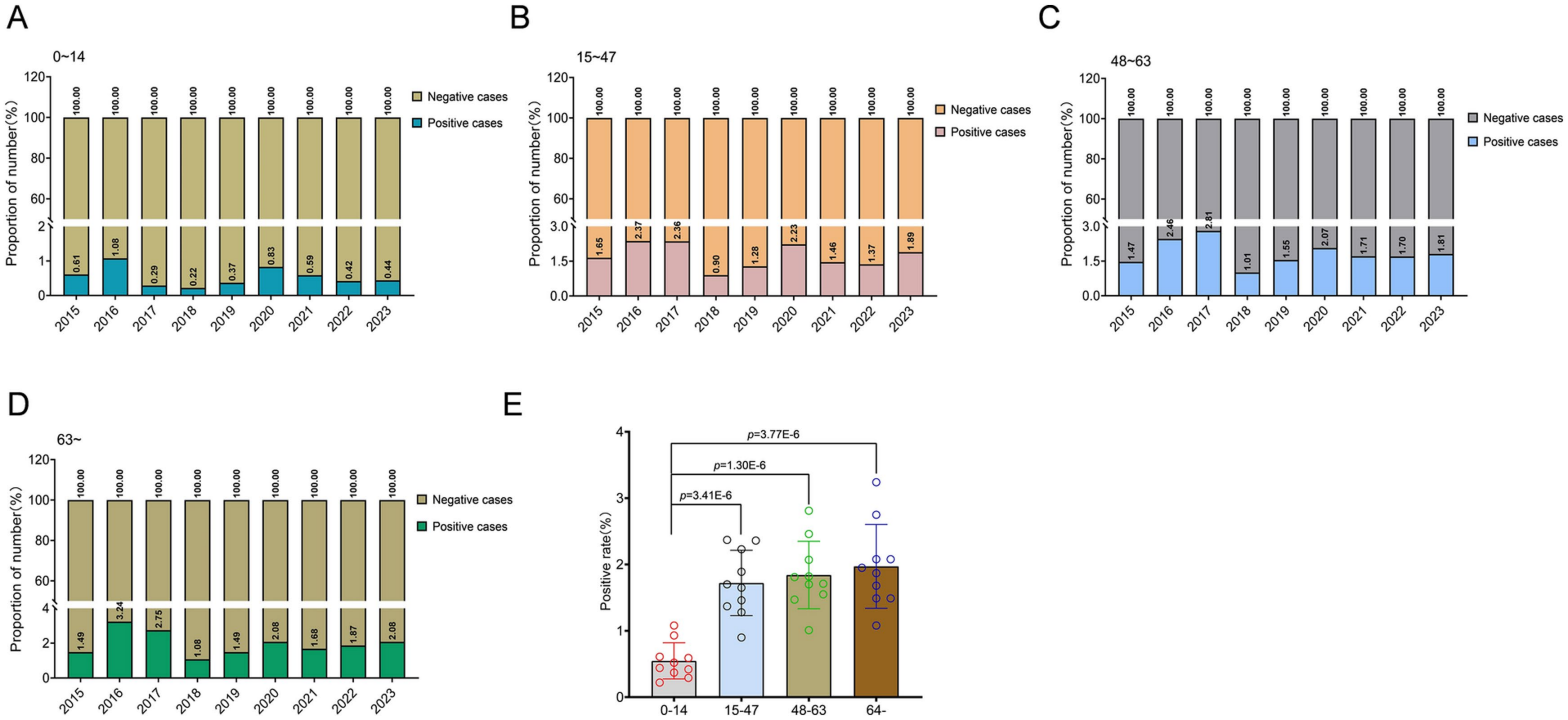
Age-specific variations in *A. baumannii* infection patterns in Guizhou Province from 2015 to 2023. **(A–D)** Epidemiological analysis of *A. baumannii* infection across different age cohorts in Guizhou province. **(E)** The distribution of age groups among patients infected with *A. baumannii*. In **(E)**, data were shown as mean ± standard deviation. Differences were assessed using two-tailed *t*-test.

Besides, a significant correlation between age and infection rate was observed (*p* < 0.001). The annual average infection rate in the four groups is: 64 (95% Cl: 42 to 87) in the age group of 0–14, 224 (95% Cl: 178 to 270) in the age group of 15–47, 236 (95% Cl: 186 to 285) in the age group of 48–63, and 248 (95% Cl: 189 to 307) in the age group over 64. However, the incidence rate of positive cases just in the 0–14 age group exhibited a significantly lower trend compared to other age groups, and this difference was statistically significant (*p* = 3.41E-6; *p* = 1.30E-6; *p* = 3.77E-6; Figures 3A–E). Among the confirmed positive patients, only 8.3% were infected within the age group of 0–14, while individuals aged 15–47, 48–63, and over 64 accounted for 29.0, 30.5, and 32.2%, respectively. The prevalence among individuals aged 0–14 remained relatively low. In 2017, only 3.1% of positive cases were in the 0–14 age group, while in 2016, individuals aged over 64 represented 35.1% of positive cases.

### *Acinetobacter baumannii* in ICUs and antibiotic resistance

**Figure 4:**
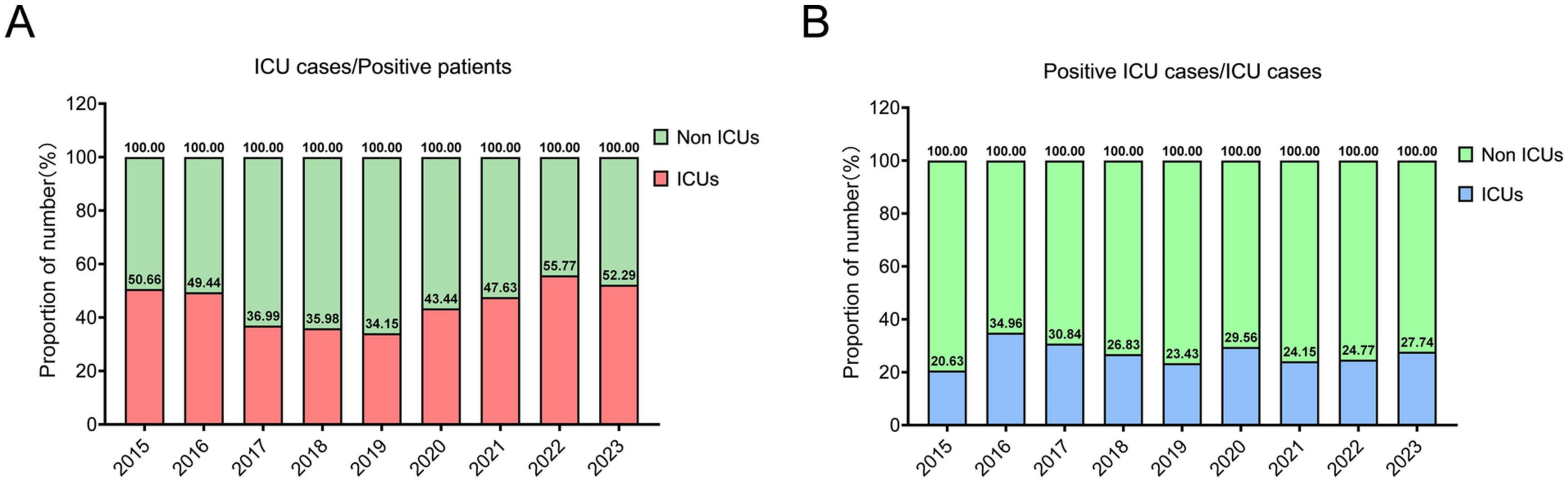
The epidemiological pattern of *A. baumannii* infection within the intensive care units (ICUs). **(A)** The ratio of ICU patients to the total number of positive patients. **(B)** The prevalence of *A. baumannii* infection among patients in the intensive care unit (ICU).

Of all confirmed *A. baumannii* cases, 45.77% occurred in ICU patients. The observed ICU incidences in 2015, 2022, and 2023 were remarkably high, with values of 50.66, 55.77, and 52.29% respectively, all surpassing the threshold of 50% (Figure 4A). The number of positive infections in ICUs exhibited a consistent decline from 2017 to 2019, with only 34.15% recorded in 2019, followed by a subsequent upward trend. Meanwhile, the prevalence of *A. baumannii* infections among ICU patients is remarkably high, reaching 27.05%. Particularly in 2016 and 2017 (Figure 4B).

The majority of the tested antibiotics here exhibited limited efficacy against *A. baumannii*. The statistical results indicate that a significant proportion of the subjects infected with *A. baumannii*, ranging from 80% to even 95%, exhibit resistance toward antibiotics such as ampicillin, cefotetan, and cefazolin. However, piperacillin, gentamicin, and amikacin demonstrate ineffectiveness in over 50% of patients. The antibiotic Polymyxin B is widely recognized as a potent and broad-spectrum agent, exhibiting remained effective against *A. baumannii* in infected patients. Among the 3,556 subjects tested positive, infection control was achieved in 3456 individuals, underscoring the promising potential of polymyxin B for future treatment strategies targeting *A. baumannii* (Table 2).

**Table 2:** The antibiotic resistance profile of *A. baumannii* against commonly prescribed medical antibiotics.

Antibiotic	Resistant (n)	Susceptible (n)	Intermediate (n)	Susceptible-dose dependent (n)	Total (n)	Resistance rate
Ampicillin	2,306	2	1	0	2,309	99.87%
Piperacillin/Tazobactam	1871	424	103	0	2,398	78.02%
Ceftazidime	4,706	890	184	0	5,780	81.42%
Ciprofloxacin	4,641	1,111	28	0	5,780	80.29%
Ceftriaxone	4,742	143	897	0	5,782	82.01%
Cefotetan	2,278	12	0	0	2,290	99.48%
Cefazolin	2,290	3	0	0	2,293	99.87%
Cefepime	4,709	1,013	55	0	5,777	81.51%
Gentamicin	3,832	1,505	443	0	5,780	66.30%
Imipenem	4,650	1,121	8	0	5,779	80.46%
Levofloxacin	2,608	1,203	1967	0	5,778	45.14%
Ampicillin/Sulbactam	4,540	1,065	171	0	5,776	78.60%
SMZ	3,774	1805	179	0	5,758	65.54%
tobramycin	3,932	1776	67	0	5,775	68.09%
Aztreonam	2,316	4	3	0	2,323	99.70%
Minocycline	1,205	2059	1,261	0	4,525	26.63%
Cefoperazone/sulbactam	2,463	574	1,122	0	4,159	59.22%
Polymyxin B	95	3,456	5	0	3,556	2.67%
nitrofurantoin	1,457	6	2	0	1,465	99.45%
amikacin	2,369	863	45	0	3,277	72.29%
Tigecycline	832	338	355	0	1,525	54.56%

## Discussion

The emergence and dissemination of *A. baumannii* in healthcare settings have become a significant public health concern (34, 35). This opportunistic pathogen has gained notoriety due to its capacity to induce severe infections, its persistence in hospital environments, and, most alarmingly, its rapid acquisition of antimicrobial resistance. The present study is based on the documented cases of *A. baumannii* infection within the healthcare system of Guizhou province from 2015 to 2023. A total of 6,944 positive cases of *A. baumannii* infection were identified among 460,620, resulting in an overall low positivity rate of 1.51% examined patients. Among the infection risk factors, age and gender have been identified as predominant contributors. Moreover, the incidence of *A. baumannii* infections in the ICUs remains elevated, presenting a substantial challenge for clinical management. Apart from polymyxin B, most commonly employed antibiotics in clinical practice demonstrate limited efficacy, thereby complicating infection prevention and control strategies within the ICU environment.

Throughout the entire duration of the statistical period, the prevalence of *A. baumannii* infections within the entire healthcare system of Guizhou is relatively low; however, the overall detection rate remains high. Among hospitalized patients infected for various reasons, the incidence of *A. baumannii* infections is notably elevated, highlighting its significant infectivity and potential susceptibility in immunocompromised populations. There is a notable surge in infection rates during 2016 and 2017, followed by a subsequent decline, suggesting the probable occurrence of localized outbreaks of *A. baumannii* within these 2 years. This might be attributed to inadequate medical prevention and control measures, or abnormal changes in the regional environmental climate facilitating the dissemination of pathogens. The seasonal distribution of *A. baumannii* infection reveals a higher incidence of susceptibility during warm spring and summer months, while infection events are relatively fewer in winter. This observation also suggests the potential preference of *A. baumannii* for high temperature environment. Despite a discernible seasonal preference for infection, no statistically significant difference was observed across the entire study period. Previous studies have indicated that *A. baumannii* may exhibit seasonal preferences; however, the findings presented here differ, potentially due to regional selection pressures. The seasonal climate changes in Guizhou might not substantially facilitate its transmission. Certainly, the necessity of intensifying pathogen detection and epidemic prevention measures were also needed during vulnerable periods, while concurrently providing enhanced care for susceptible populations.

Notably, the proportion of male individuals among infected patients is significantly higher than that of females, which implies that males are more prone to becoming susceptible populations. The data encompassing the past 9 years unequivocally highlight this striking disparity. Gender has emerged as a significant risk factor for *A. baumannii* infection. This could potentially be attributed to disparities in the working environment, internal physiological conditions, and even genetic variations between males and females. However, the question of whether this sex predisposition stems from environment, physiological or genetic factors remains to be fully elucidated through more in-depth experimental investigation. Age emerges as a pivotal risk factor for *A. baumannii* infection. Among the individuals who tested positive, the prevalence of patients aged 0–14 is significantly lowers compared to other age groups. The incidence of infection is slightly higher in the population aged over 64 compared to other age groups. A positive correlation between infection events and increasing age is observed. The older adult population is particularly vulnerable to *A. baumannii* infection, potentially attributed to immunosenescence and heightened healthcare exposure. The elevated incidence of comorbidities and more frequent visits to medical institutions have significantly heightened the susceptibility risk.

The ICUs are equipped with isolation facilities and specialized equipment to provide optimal care, comprehensive treatment, and ancillary services for critically ill or comatose patients (36–38). The study observed a notable concentration of infected individuals in ICUs, including both community-onset and hospital-acquired cases. As commonly acknowledged, ICU equipment and the surrounding environment undergo regular sterilization and disinfection; however, this scenario still gives rise to instances of *A. baumannii* infection, highlighting its formidable resistance. There have been reports indicating the ability of *A. baumannii* to form biofilms on medical devices, including catheters, tracheas, and intubation equipment, thereby posing significant challenges in eradication and elevating the risk of nosocomial infections (39, 40). Here, out of over 40 antibiotics investigated, only polymyxin B demonstrates potent inhibitory activity against *A. baumannii* (97.33%), while the majority exhibit limited or no efficacy. The formidable drug resistance and intricate mechanisms of resistance pose a significant medical challenge in combating *A. baumannii* (41, 42). Carbapenem resistance in *A. baumannii* limits the efficacy of imipenem and meropenem in ICU settings, contributing to treatment challenges. Furthermore, with the evolution of drug-resistant strains, the efficacy of individual polymyxin B agents may be limited. Consequently, there is an urgent imperative to discover or develop more potent pharmaceutical formulations.

This study is subject to several limitations. Considering China’s expansive territory, the nation’s climate, environment, and other conditions display a remarkable spectrum of diversity that may not be adequately captured by this localized focus. Moreover, the geographical restriction might limit generalizability, such as changes in air quality attributable to altitude or changes in temperature attributable to seasonal variations. Besides, these variations can exhibit profound differences on a global scale. Furthermore, in the realm of data collection and analysis, age and gender were identified as salient risk factors for *A. baumannii* infection. However, this study did not delve into whether these factors operate independently or synergistically to influence infection rates. This oversight could potentially undermine the formulation of subsequent epidemic prevention policies and the identification of key populations that warrant heightened attention. Age and gender may act independently or synergistically, and multivariable analysis or interaction models may be needed established as a potential future approach for *A. baumannii* infection analysis. The dependence on historical data extracted from medical records might introduce potential biases, whereas the lack of molecular information significantly hampers our capacity to precisely elucidate the intricate transmission dynamics. Also, the lack of molecular data could restrict conclusions on transmission or resistance mechanisms, which may impose certain limitations on the development of more effective antibiotics and molecular drugs via molecular medical engineering. A more comprehensive integration of molecular biology tools will generate richer, more robust, and profoundly insightful scientific data. In order to achieve a more comprehensive understanding of the epidemiology of *A. baumannii*, it is advisable to complement traditional epidemiological statistical analyses with molecular biology methods, such as genomics. This integrated approach not only highlights regional infection differences but also offers deeper insights into the molecular basis of these variations.

## Conclusion

Not only do ICUs account for the majority of positive cases, but also the significant incidence of *A. baumannii* infections among ICU-admitted patients within Guizhou province. These findings suggest that effective suppression methods for *A. baumannii*, such as antimicrobial stewardship, environmental cleaning and hand hygiene, compliance are still lacking in ICU system of Guizhou province, highlighting a significant burden. Moreover, *A. baumannii* exhibits remarkably high drug resistance to some antibiotics, such as carbapenems and aminoglycosides, and the availability of effective antibiotics in the collected data is limited (just polymyxin B). Overall, there has been limited progress in the prevention and control of *A. baumannii* in Guizhou province over the past 9 years, particularly concerning key populations, such as older adults, immunocompromised, and ICU patients. Efforts such as implementing molecular surveillance, optimizing infection control policies, and conducting multicentre studies remain insufficient or are progressing slowly. In summary, further enhancement of overall efforts toward drug development for prevention and treatment may be needed.

## Data Availability

The original contributions presented in the study are included in the article/supplementary material, further inquiries can be directed to the corresponding author.
